# Tong Luo Jiu Nao ameliorates Aβ_1–40_-induced cognitive impairment on adaptive behavior learning by modulating ERK/CaMKII/CREB signaling in the hippocampus

**DOI:** 10.1186/s12906-015-0584-9

**Published:** 2015-03-11

**Authors:** Zhe Shi, Cong Lu, Xiuping Sun, Qiong Wang, Shanguang Chen, Yinghui Li, Lina Qu, Lingling Chen, Lanlan Bu, Duanfang Liao, Xinmin Liu

**Affiliations:** Research Center for Pharmacology and Toxicology, Institute of Medicinal Plant Development (IMPLAD), Chinese Academy of Medical Sciences and Peking Union Medical College, Malianwa North Road No. 151, Beijing, 100193 China; Division of Stem Cell Regulation and Application, Hunan University of Chinese Medicine, Changsha, 410208 Hunan China; China Astronaut Research and Training Center, Yuanmingyuan West Road No. 1, Beijing, 100094 China

**Keywords:** Aβ_1–40_, Cognitive impairment, Tong Luo Jiu Nao, Reward-directed instrumental learning, ERK/CaMKII/CREB signaling

## Abstract

**Background:**

Tong Luo Jiu Nao (TLJN), a modern formula of Chinese medicine extracts on the basis of Traditional Chinese Medicine theory, has been used to treat dementia. The present study aimed to investigate its ameliorating effects on Aβ_1–40_-induced cognitive impairment in rats using a series of novel reward-directed instrumental learning (RDIL) tasks, and to determine its possible mechanism of action.

**Methods:**

Rats were pretreated with TLJN extract (0.9 and 1.8 g/kg, p.o.) for 10 daysbefore surgery, and were trained to gain reward reinforcement by lever pressing at the meantime. Thereafter, rats received a bilateral microinjection of Aβ_1–40_ in CA1 regions of the hippocampus. Cognitive performance was evaluated with the goal directed (higher response ratio) and habit (visual signal discrimination and extinction) learning tasks, as well as on the levels of biochemical parameters and molecules.

**Results:**

Our findings first demonstrated that TLJN can improve Aβ_1–40_-induced amnesia in RDIL via enhancing the comprehension of action-outcome association and the utilization of cue information to guide behavior. Then, its ameliorating effects should attribute to the modulation of ERK/CaMKII/CREB signaling in the hippocampus.

**Conclusion:**

TLJN can markedly enhance cognitions of Aβ_1–40_ microinjection animal model in adaptive behavioral tasks. It has the potential, possibly as complementary and alternative therapy, to prevent and/or delay the deterioration of cognitive impairment in AD.

## Background

Alzheimer’s disease (AD) is the most common form of dementia in the elderly which is pathologically characterized by senile plaques and neurofibrillary tangles, together with a degeneration of the neurons and synapses [1]. AD is a slowly progressive neurodegenerative disorder, with insidious onset and progressive impairment of general cognitive symptoms, such as impaired episodic memory, judgment, decision-making, and orientation [1,2]. A primary disability in learning and retaining new information is one of the initial symptoms of AD [3]. This characteristic amnestic symptom, which possibly leads to the dysfunctions in information processing, attention and executive functions, renders the patient incapable of drawing on advantages and avoiding disadvantages, not only resulting in a gradual loss of the ability to take care of themselves [4] but also an increase in social care costs [5]. However, assessing the effects on the ability to make an adaptive behavioral adjustment when facing with context changes has been neglected in screening and evaluating potential therapeutic drugs for AD. Thus, a feasible method for detecting this pivotal aspect of cognition would contribute to a more comprehensive understanding of the drugs in preclinical research. Based on this consideration, our laboratory recently introduced a series of reward-directed instrumental learning (RDIL) tasks for studying cognitions in adaptive behavior [6,7]. Instrumental conditioning, which is also called operant conditioning, is a form of associative learning through which an animal learns to modify its behavior from the foreseeable consequences [8]. It is one of the most elementary forms of adaptive behavior [9] and reflects the remarkable aspects of ability that reaches its highest form in human beings [10]. Since a behavior that has once produced a positive consequence could later produce a negative consequence. This flexibility allows rapid behavioral alterations in the face of changing consequences, conferring a survival advantage [11].

Furthermore, although the precise aetiology of AD is still less well known, beta-amyloid peptide (Aβ) has been widely accepted as a crucial pathogenic factor in disease development [12-14]. Several lines of evidence indicate that Aβ are primarily responsible for both the neuronal dysfunction and cognitive deficits, even before the appearance of overt toxicity [15-17]. Particularly in the hippocampus, where external information is processed and diverse features of experience is encoded [6], Aβ disrupt neuronal plasticity processes and long term potentiation (LTP) which are critically related to cognitive functions [14,18,19]. Moreover, substantial evidence demonstrates that multiple neurotransmitters, cell surface protein receptors and intracellular signal transductions, including acetylcholine (Ach), glutamate (Glu), muscarinic acetylcholine receptors (mAChR), N-methyl-D-aspartic acid (NMDA) receptors, extracellular signal-regulated kinase (ERK), Ca^2+^/calmodulin-dependent protein kinase II (CaMKII) and cAMP response element-binding protein (CREB) have been implicated in mediating Aβ induced cognitive dysfunction [20-23].

In addition, accumulating studies have recently shown that herbal preparations may provide a prospective alternative in the treatment of dementia for their better compliance and lower side effects. Tong Luo Jiu Nao (TLJN) pill is a modern formula of Chinese medicine extracts on the basis of traditional Chinese medicine theory which has been applied in dementia treatment for decades [24]. Previous evidence from both clinical and experimental researches has demonstrated that TLJN is curative in treating ischemic cerebral stroke, vascular dementia [25,26] and AD [24]. It has been reported that TLJN could ameliorates local ischemia and AD in terms of spatial learning and memory through modulating cell survival, angiogenesis and neurogenesis [24-26]. However, no attempt has been made as yet to determine its therapeutic effects on adaptive behavior learning in Aβ_1–40_ microinjection animal model and consequent influence on memory-related molecules. Therefore, the aim of the present research was to investigate the ameliorating effects of TLJN on RDIL in memory impaired rats and determine its influence on memory-related biochemical parameters and molecules. Our findings first demonstrated that TLJN can improve Aβ_1–40_-induced amnesia in RDIL via enhancing the comprehension of action-outcome association and the utilization of cue information to guide behavior. Then, its ameliorating effects should attribute to the modulation of ERK/CaMKII/CREB signaling in the hippocampus.

## Methods

### Animals

Sixty male Wistar rats (Vital river, Beijing, China), weighing 300–320 g at the beginning of the experiments, were housed 4 to a cage with lights on from 7:00–19:00. The rats were maintained at 85% of an adjusted ad libitum body weight throughout the duration of the study by a controlled diet of standard laboratory chow (16 g/day). Once training began, they were fed each day after the training sessions, and had free access to water while in their own cage each day 30 min after the training sessions with water freely available while in their own cages. All rats, regardless of group, received the same handling and feeding during this phase of the experiment. The experiment was carried out according to the “Principles of Laboratory Animal Care” (NIH publication No.86–23, revised in 1996) and P. R. China legislation for the use and care of laboratory animals. All efforts were made to minimize animal suffering during experiments. The protocols were approved by the committee for the Care and Use of Laboratory Animals of IMPLAD, CAMS & PUMC, China (No. 2011032).

### Drugs preparation and administration procedure

**Figure 1:**
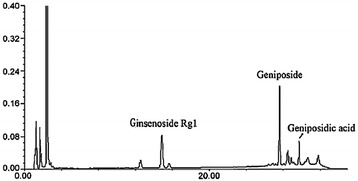
**HPLC analysis of TLJN.** The concentrations of geniposide, ginsenoside and geniposidic acid were equivalent to the corresponding TLJN content, and 7.7 mg/ml TLJN contained 4.95 mg/ml, 1.02 mg/ml and 1.73 mg/ml of geniposide, ginsenoside and geniposidic acid, respectively.

The TLJN is produced by Heyi Biosciences Limited Company, Tianjin, China (Lot No: 20110321, kindly supplied by Prof. Li Pengtao in the School of Preclinical Medicine, Beijing University of Chinese Medicine). In Briefly, TLJN are extracted from Panax notoginseng and Gardenia jasminoides. The amounts of Panaxnotoginseng (5 g) and Gardenia jasminoides (8.5 g) used were based on knowledge gained from clinical practice. The preparation of TLJN was according to the procedure which has been previously described in detail [24]. The total concentration of the active components of TLJN extract was 7.7 mg/g based on data from processing and stability studies, the active ingredients of TLJN were consisted of geniposide (64.28%), geniposidic acid (13.25%) and ginsenoside Rg1 (22.47%), which were identified with a high performance liquid chromatography (HPLC) method (see Figure 1) [26].

Amyloid β Protein Fragment 1–40 (Aβ_1–40_), which was purchased from Sigma-Aldrich (St. Louis, MO, USA), was dissolved in sterile double-distilled water at a concentration of 5 μg/μl and incubated at 37°C for 7 days prior to use. Donepezil hydrochloride (Aricept), which was purchased from Eisai (Ibaraki, Japan), was dissolved in distilled water.

**Figure 2:**
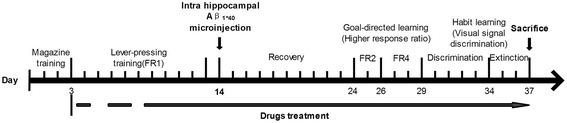
**Experimental manipulation of rats during the course of behavioral testing.**

Rats were assigned to five groups (n = 12 each) in a quasirandom manner. Initial random group assignments were adjusted using baseline magazine training performance to control for a response bias. After three days magazine training, rats in each group respectively received orally water (sham and Aβ_1–40_ groups), donepezil hydrochloride (DNP group, 3 mg/kg) and TLJN (TLJN min group, 0.9 g/kg; TLJN max group, 1.8 g/kg) until the end of the behavioral test (see Figure 2).

### Reagent

Antibody for mAChR M1 (H-120) was purchased from Santa Cruz Biotechnology (Santa Cruz, USA). Antibodies for Phospho-NMDAR1 (Ser890), NMDAR1 (D65B7), Phospho-NMDAR2B (Tyr1070), NMDAR2B, Phospho-CaMKII (Thr286), CaMKII (pan), Phospho-p44/42 MAPK (Erk1/2) (Thr202/Tyr204), p44/42 MAPK (Erk1/2) (137 F5), Phospho-CREB (Ser133) and CREB (48H2) were obtained from Cell Signaling Technology (Cell Signaling, USA). Anti-GAPDH and secondary antibodies purchased from ZSGB-Bio (ZSGB-Bio, China).

### Apparatus

Behavioral testing was conducted in four operant chambers (Xin Hai Hua Yi Instrument Co., Beijing China) which has previously been described in detail [6]. Herein, we make a brief introduction. The operant chambers were placed in sound-attenuated room. Each one fitted with a recessed dipper magazine and a retractable lever. Three color LED signal lights were located above the lever. The chambers could be illuminated by a LED house light located on the ceiling. An infrared beam emission and acceptance device was fixed on the side wall of the magazine to record the nose poke activity. Ventilation and a masking noise were provided by an exhaust fan.

### Surgical procedures

Rats were anesthetized with 10% chloral hydrate diluted in physiological saline (3.5 ml/kg, IP) and placed into a stereotaxic apparatus (Benchmark, USA) with head held horizontally. A midline incision was then made into the scalp and the scalp was retracted. Small holes were then drilled into the skull above the injection sites using a dental burr. The stereotaxic coordinates to conduct a bilateral microinjection in the CA1 region of the hippocampus (anterior-posterior (AP) = −3.3 mm, medial-lateral (ML) = ±2.0 mm from the bregma and dorsal-ventral (DV) = 3.0 mm from cerebral dura mater) were standardized from the stereotaxic atlas of Paxinos and Watson [27]. A flatted-tipped Hamilton syringe lowered into the bilateral hippocampus and either 5 μl of saline (sham group) or Aβ_1–40_ was delivered at a rate of 1 μl per min. Following the injection, the needle was kept in place for 5 min prior to its slow extraction. Rats of the sham group were infused with the vehicle only. After surgery, animals were placed in heated chambers in a darkened room and allowed to recover with free access to food and water. The experiment was continued after 10 days of recovery as follows.

### Behavioral task

#### Magazine training

When subjects were at 85% of their ad libitum weight, all of them were habituated to the operant box over three consecutive 20 min sessions in which the reward (approximately 0.2 ml) was delivered daily indiminishing amounts (30, 25 and 20 drops) on a Random Time (RT) (40-s, 48-s and 60-s) schedule with the lever withdrawn. Each session began with the onset of the house light and terminated with its offset after 20 min. During the magazine process, rats in the operant chamber were only exposed to a blue cue light and white noise. The blue cue light was simultaneously illuminated with the appearance of the reward which was an 8% (*m/v*) solution of sucrose in distilled water that was prepared daily before each session.

### Lever pressing response acquisition

After three sessions of magazine training, the lever was inserted. Rats were trained to freely press the lever for the sucrose reward under continuous reinforcement schedule (Fixed-ratio 1, FR1). Ten consecutive 30 min sessions were conducted. The blue cue light was simultaneously illuminated for 10 sec following the acquisition of reward. The daily session was terminated after either 50 rewards or 30 min, whichever came first.

### Post-surgery test

Subjects underwent the intra hippocampal microinjection of Aβ_1-40_ after 10 days of lever pressing, and after 10 days recovery from the surgery, the following procedures were conducted.

### Goal-directed learning/contingency degradation (Higher Fixed Ratio schedule)

After recovery, the subjects were trained to press the lever at a higher frequency of response under the FR2 and FR4 schedule, meaning that during the session, every 2 or 4 pressings of the lever resulted in one reward delivery. We further defined that rats could only obtain a reward by finishing consecutive responses within 2 sec with the FR2 schedule and 3 sec with the FR4 schedule. Six consecutive 30 min sessions were conducted. The blue cue light was illuminated following each successful lever pressings and left on for 10 sec after the reward was presented. The process was terminated when the subjects earned 50 rewards during a 30 min session or when timed out [6,7].

### Habit learning (Signal discrimination and Extinction)

A: Discrimination of conditioned cue signaling
  The training period lasted 5 days. During the training course, blue and red cue signals were alternately turned on in 120 s period. Fourteen sessions were conducted daily. The blue light (S^+^) served as a reward predictor while the red light (S^−^) was associated with a non-rewarded consequence. Rats were trained to lever press in response to the alternating visual cues. They could earn one reward after one lever pressing in S^+^ phase, and this action was considered a correct response [6,7].
B: Extinction
  Testing under the signal discrimination schedule was followed by three 20 min extinction sessions. Rats were exposed to the signal discrimination environment but experienced no scheduled consequences in response to correct lever pressing [6,7].

### Biochemical analysis

Immediately upon completion of the extinction session, the rats were anesthetized and then sacrificed. Their brains were rapidly removed, and the hippocampuses were dissected out on ice. The concentration of acetylcholine (Ach) and glutamate (Glu) was determined by a LC-MS/MS method. The tissues were weighed and homogenized in ice-cold 0.2% aqueous formic acid, then mixed with 0.2% formic acid in acetonitrile for protein precipitation. After centrifugation at 12000 rpm for 10 min at 4°C, an aliquot of the supernatant (200 μl) was collected and mixed with 20 μl of internal standard solution (300 μg/ml DHBA). Fifty micro-liters of the mixture were injected into a LC-MS/MS system for assay. The LC-MS/MS instrument was equipped with an Agilent 1200 HPLC system (Palo Alto, CA, USA) and an Applied Biosystem 3200 Q-Trap mass spectrometer (Foster City, CA, USA) with an electrospray ionization source. The mobile phase consisted of 6 mM ammonium formate in acetonitrile-water (67.5:32.5, pH 5.50) with a flow rate of 200 μl/min. The neurotransmitters and internal standard were detected in multiple reaction monitoring mode. Ratios of the peak areas of the analyte versus the internal standard were used to quantify the neurotransmitter concentrations.

### Western blotting

The hippocampuses was promptly dissected out on ice and homogenized in ice-cold RIPA buffer containing a protease inhibitor cocktail for 30 minutes. After centrifugation for 10 min at 4°C and 12,000 rpm, supernatants were divided into eppendorf tubes and stored at-20°C until required for protein assay, which was performed using Pierce BCA protein assay kits (Thermo scientific, USA). For western blot analysis, mixed one part of sample loading buffer (Applygen Tech Inc., China) with 4 parts of tissue protein and then boiled the mixture at 100°C water bath for 10 minutes. Denatured proteins were separated by 10% SDS-PAGE gel (CWBIO, China) electrophoresis for 1 h at 80 V then shifted to 100 V for another 2 h. Thereafter transferred to a 0.45 μmpolyvinylidene-difluoride (PVDF) membranes for 50 minutes at 100 V. Membranes were then washed for 3 × 10 min in 0.1% Tween-20 PBS (TBST) between each of following steps: 1 h block in 5% non-fat milk, over-night incubation at 4°C with primary antibodies. The immunoreactive bands were visualized by using secondary antibodies and ECL chemiluminescence detection kit (CWBIO, China).

### Statistical analysis

All analyses were performed using SPSS version 16.0 (Chicago, IL, USA). Values were expressed as means ± standard error of the mean (SEM), and statistical significance was set at *p* < 0.05 in all of the evaluations. The results of the analysis of these data were only reported when a significant difference was observed.

The data were analyzed with one-way or repeated measures analysis of variance (RM ANOVA) where statisticallyappropriate. RM ANOVA were used to analyze differences between groups throughout the behavioral test with days as the within-subject variable and different treatment groups as the between-subject variable. Mauchley’s test was used to evaluate the sphericity of the within-subject effects, and when necessary, the Greenhouse–Geisser was applied to adjust the degrees of freedom. When significant effects were detected, post hoc multiple pairwise comparisons were made using the LSD comparisons test after ANOVA. One-way ANOVA were used to test differences between groups in biochemistry and molecular biology tests.

## Results

### Magazine training

Before the instrumental task was conducted, magazine training was carried out first to insure that all the subjects could make the simplest Pavlovian S-O association, to train the rats about the location of the reward, to teach them the signals associated with reward delivery (visual stimuli in the present experiment), to keep the rats aroused and to promote exploration [28]. The sucrose reward was paired with the cue signal which resulted in exploration of the magazine. There were no significant differences in Nose Pokes (NPs) activities (results not shown) among the groups. This implied that when the training started, the initial incentive motivation aroused by the reward substance was at the same level in all groups.

### Acquisition of the basic instrumental response (lever pressing)

The LPs and LP/NP ratios increased progressively, while the NPs decreased during the training days in all groups. The results indicated that all the subjects could shape instrumental conditioning across training days. The established Pavlovian Signal-Outcome (S-O) association was shifted to an Action-Outcome (A-O) association in the lever-pressing training course. Nonetheless, drug treated groups did not display a nootropic effect in normal rats. Furthermore, no significant differences in the levels of LPs, NPs and LP/NP ratios were evident among the groups at the end of the testing periods (results not shown). It was clear that the primary instrumental response acquired before surgery was not affected by drug treatment in normal rats.

TLJN can improve behavioral performances in goal-directed learning (higher rate of response schedule) in post-training surgically manipulated animals.

**Figure 3:**
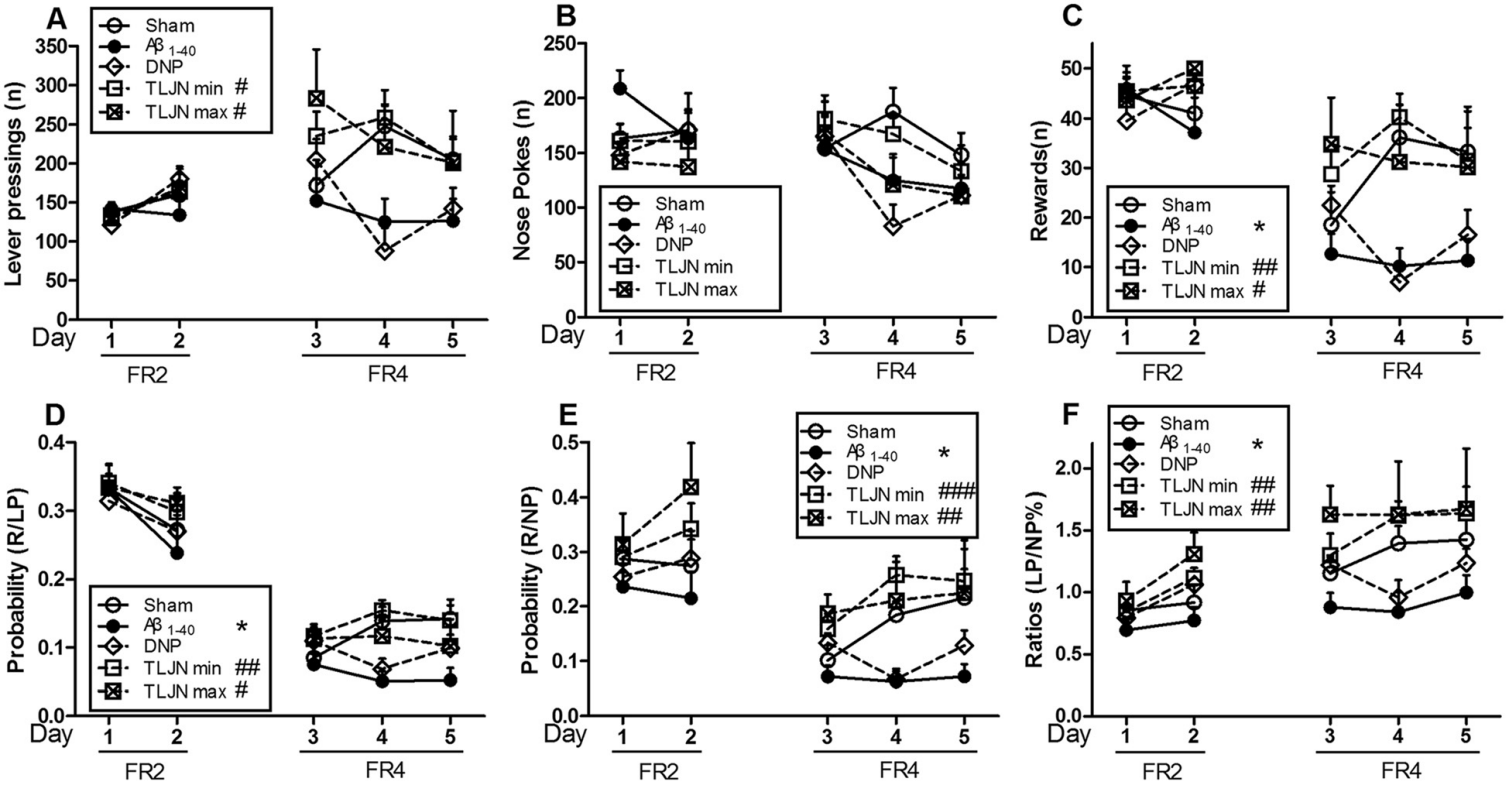
**Behavioral performance in goal-directed learning (higher rate of response schedule) in post-training surgically manipulated animals.** Subpart **(A)** is the number of daily LPs; subpart **(B)** is the number of daily NPs; subpart **(C)** is the number of Rs earned per day; subpart **(D)** is the daily ratios of P(R/LP); subpart **(E)** is the daily ratios of P(R/NP) and subpart **(F)** is the daily ratios of LP/NP. Data are expressed as means ± SEM, n=10.Significant differences *p<0.05 compared with the sham; #*p*<0.05, ##*p*<0.01, ###*p*<0.001 compared with the Aβ_1-40_.

We used RM to analyze the interaction effects between groups and days of training. As shown in Figure 3, there was no significant change in any parameter on employing the FR2 schedule. Following the FR4 schedule, the Aβ_1–40_ model group significantly showed a tendency of decreased Rs, P (R/LP), P (R/NP) and R (LP/NP) values (Figure 3(C) - (E)) compared with sham group. TLJN treated groups (0.9 and 1.8 g/kg, p.o.) performed an ameliorating effect on Aβ_1–40_-induced cognitive deficit in goal-directed learning task. However, DNP treated rats display a limited extent of therapeutic effect in this stage of adaptive behavior learning.

TLJN can improve behavioral performances in habit learning (visual signal discrimination and extinction task) in post-training surgically manipulated animals.

#### A: Discrimination session

**Figure 4:**
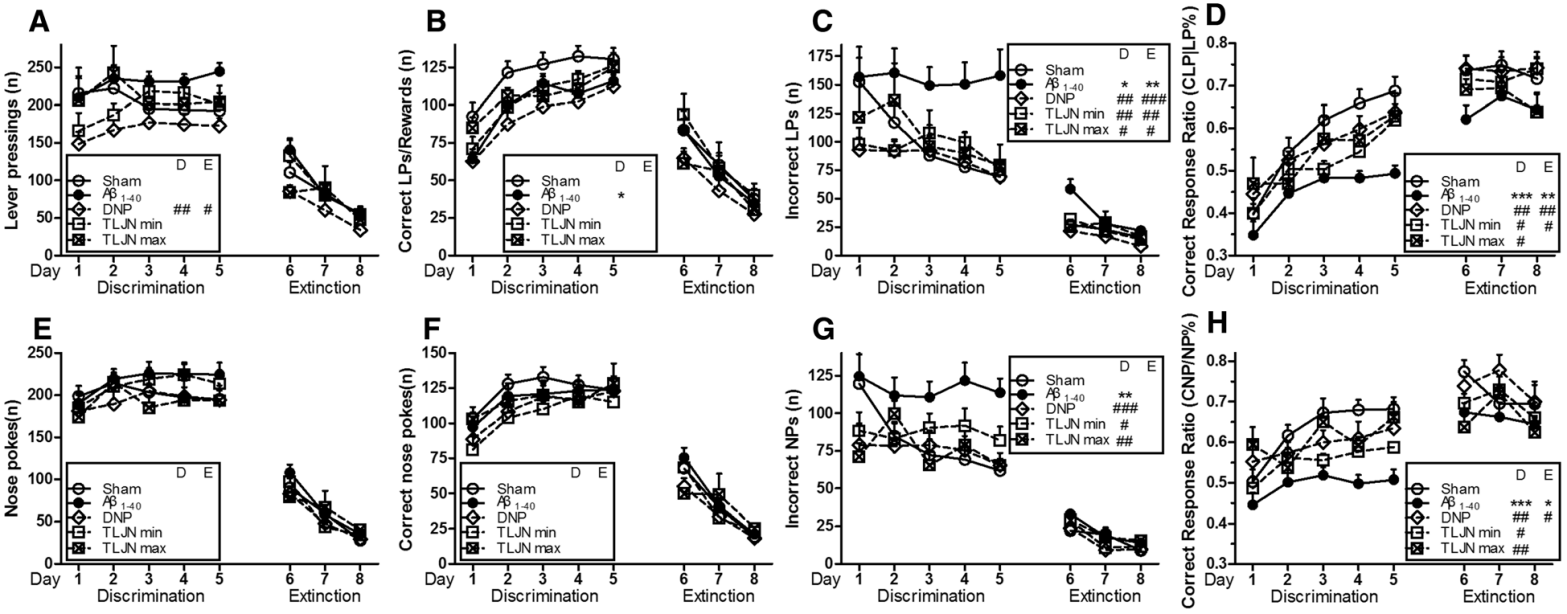
**Behavioral performance in habit learning (visual signal discrimination and extinction task) in post-training surgically manipulated animals.** Subpart **(A)** is the number of LPs per day; subpart **(B)** is the number of CLPs per day; subpart **(C)** is the number of ILPs per day; subpart **(D)** is the CLPR (CLP/LP) per day; subpart **(E)** is the number of NPs per day; subpart **(F)** is the number of CNPs per day; subpart **(G)** is the number of INPs per day; subpart **(H)** is the CNPR(CNP/NP). All data are expressed as means ± SEM, n=10. Significant differences *p<0.05, **p<0.01, ***p<0.001 compared with the sham; #*p*<0.05, ##*p*<0.01, ###*p*<0.001 compared with the Aβ_1-40_.

As shown in Figure 4, no significant differences in NPs and CNPs were found. All parameters either increased or decreased progressively during the training days in all groups except for LPs. It demonstrated that the ability to identify an S-R association could be progressively enhanced under a stable level of lever-pressing activity. The Aβ_1–40_-treated group performed significant fewer CLPs, lower CLPR, CNPR and more ILPs, INPs than sham group, indicating that the Aβ_1–40_-administrated rats unable to adjust their responses to the cue reflecting a correct association with the reward outcome. All the drug treated groups, including TLJN min, TLJN max and DNP, displayed a marked ameliorating effect in the stage of habit learning.

#### B: Extinction session

The extinction session was carried out in the absence of the reinforce to probe the nature of the memory. Significant differences were found in LPs (DNP vs. Aβ_1–40_, F (4,45) = 1.347, *p* = 0.029), ILPs (Aβ_1–40_ vs. Sham, F (4,45) = 2.240, *p* = 0.002; DNP vs. Aβ_1–40_, *p* < 0.001; TLJN min vs. Aβ_1–40_, *p* = 0.004; TLJN max vs. Aβ_1–40_, *p* = 0.015), CLPR (Aβ_1–40_ vs. Sham, F (4,45) = 3.658, *p* = 0.005; DNP vs. Aβ_1–40_, *p* = 0.003; TLJN min vs. Aβ_1–40_, *p* = 0.011) and CNPR (Aβ_1–40_ vs. Sham, F (4,45) = 2.523, *p* = 0.049; DNP vs. Aβ_1–40_, *p* = 0.013) during the extinction test. The results further confirmed the curative effect of TLJN should attribute to the improved comprehension of the causal association between visual signal and specific responses.

### TLJN can increase Ach and reduce Glu content in the hippocampus

**Figure 5:**
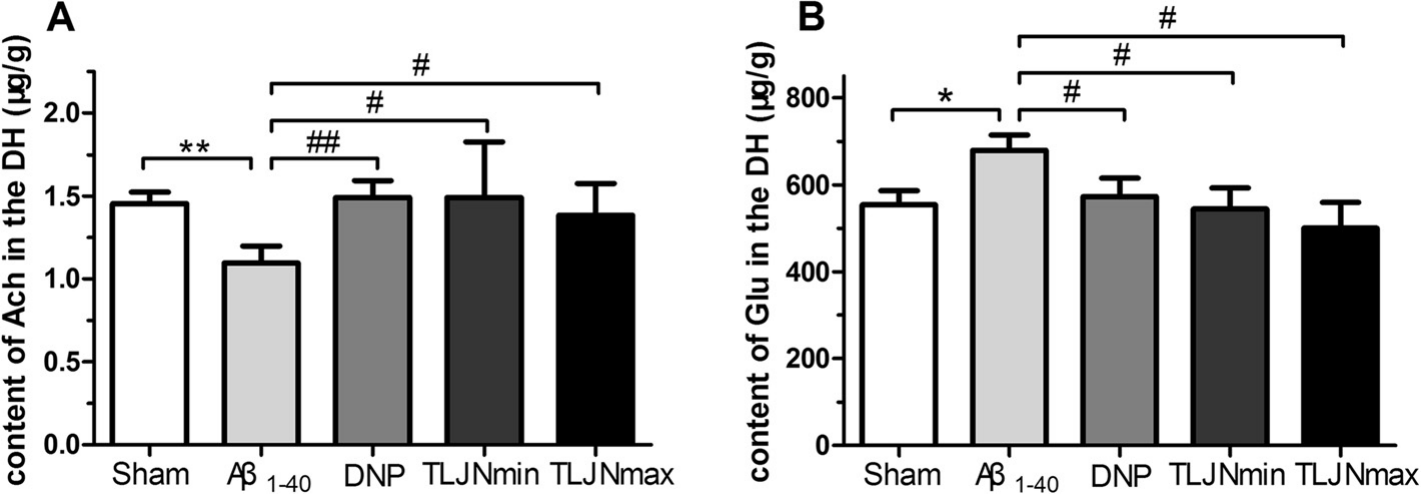
**Alteration of hippocampal acetylcholine and glutamate levels of post-training surgically manipulated animals after the behavior procedure.** Subpart **(A)** is the content of Ach in the hippocampal; subpart **(B)** is the content of Glu in the hippocampal. All data are expressed as means ± SEM, n=6. Significant differences *p<0.05, **p<0.01 compared with the sham; #*p*<0.05, ##*p*<0.01 compared with the Aβ_1-40_.

We tested neurotransmitter levels in the hippocampus after behavioral procedures were completed. Differences between groups were analyzed with one-way ANOVA. As shown in the subpart (A) of Figure 5, the TLJN min, TLJN max and DNP treated groups showed a markedly increased Ach levels in the hippocampus. The Ach level was significantly different on comparing Aβ_1–40_ vs. sham (F (4,26) = 2.166, *p* = 0.006) group. The TLJN min (*p* = 0.043), TLJN max (*p* = 0.035) and DNP (*p* = 0.004) treated groups showed a markedly increased Ach levels vs. Aβ_1–40_ group in the hippocampus. Moreover, as shown in the subpart (B) of Figure 5 the TLJN min, TLJN max and DNP treated groups showed a markedly decreased Glu levels. The Glu level was significantly different on comparing Aβ_1–40_ vs. sham (F (4,26) = 2.362, *p* = 0.017) group. The TLJN min (*p* = 0.020), TLJN max (*p* = 0.016) and DNP (*p* = 0.030) treated groups showed a markedly decreased Glu levels vs. Aβ_1–40_ group in the hippocampus.

TLJN can improve the expression levels of memory-related molecules in the hippocampus

**Figure 6:**
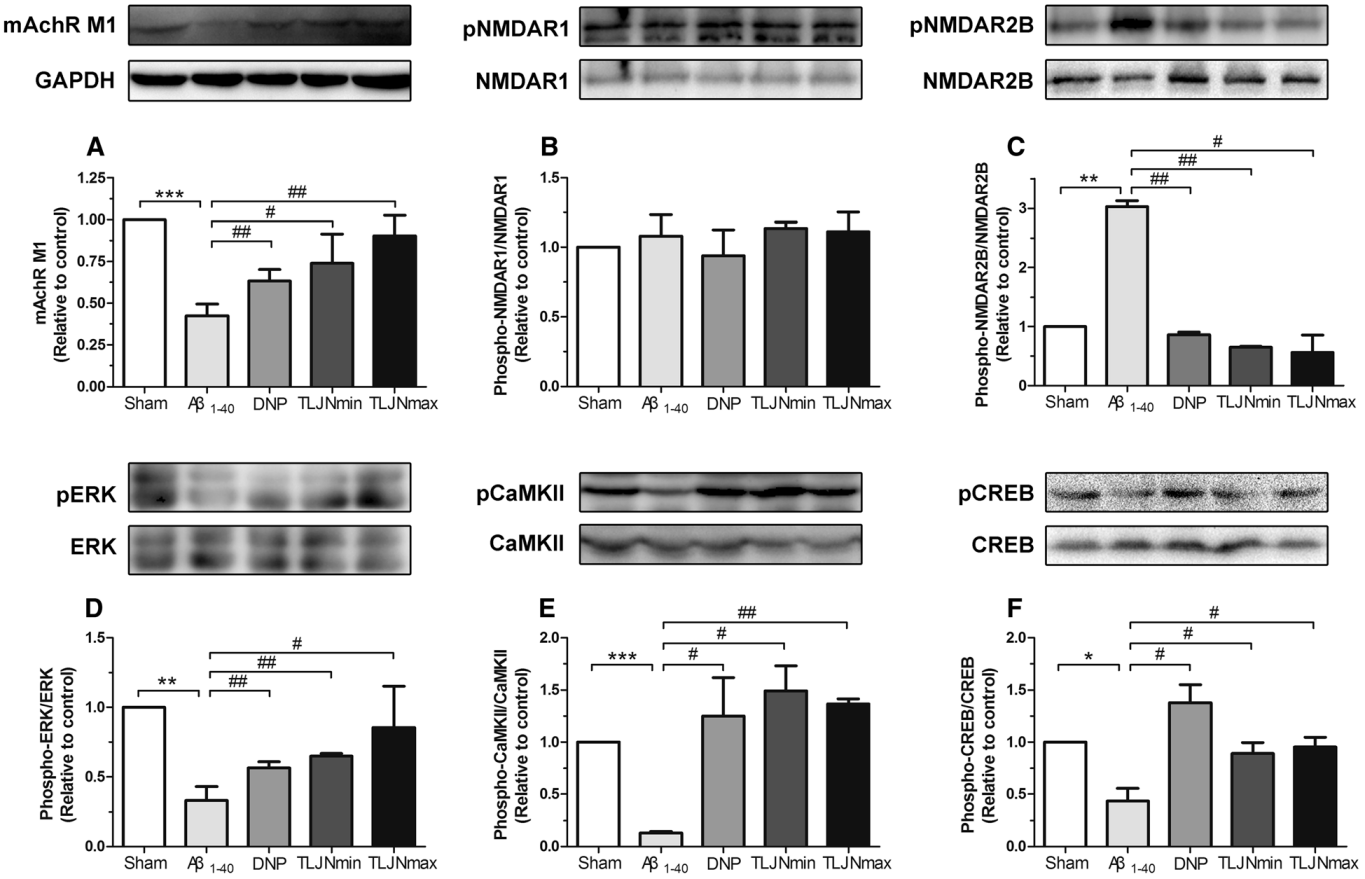
**Effects of drugs administration on the expression levels of memory-related molecules in the hippocampus.** Subpart **(A)** is the expression levels of mAchR M1 receptor; subpart **(B)** is the expression levels of phosphor-NMDAR1 and NMDAR1 proteins; subpart **(C)** is the expression levels of phosphor-NMDAR2B and NMDAR2B proteins; subpart **(D)** is the expression levels of phosphor-ERK1/2 and ERK1/2 proteins; subpart **(E)** is the expression levels of phosphor-CaMKII and CaMKII proteins; subpart **(F)** is the expression levels of phosphor-CREB and CREB proteins;. All data are expressed as means ± SEM, n=3. Significant differences *p<0.05, **p<0.01, ***p<0.001; compared with the sham; #p<0.05, ##p<0.01 compared with the Aβ_1–40_.

To investigate how TLJN treatment effect on Aβ_1–40_-induced cognitive dysfunction, Western blotting analysis was used to measure alterations of memory-related molecules after behavioral tests. As shown in Figure 6, Aβ_1–40_ group displayed marked declines in mAchR M1 expression, p-ERK1/2/ERK1/2, p-CaMKII/CaMKII and p-CREB/CREB ratio, and an increase in p-NMDAR2B/NMDAR2B ratio compared with control group. However, no obvious alteration was found in p-NMDAR1/NMDAR1 ratio. Moreover, the results revealed that TLJN (0.9 and 1.8 g/kg) together with DNP (3 mg/kg) could significantly reverse Aβ_1-40_ induceddisorders in memory-related molecules expression.

## Discussion

The hippocampus is essential for encoding diverse features of the animal’s experience such as spatial locations, landmarks, visual features of the environment, goal locations, conditioned stimuli, and sequences of events [6]. Taking into account the impairment of contextual memory acquisition and retention [29-31], our previous findings demonstrated that the microinjection of Aβ_1–40_ into the CA1 region of the hippocampus did render the rats unable to process explicit information for guiding behavior rather than influencing the behavior per se in RDIL [6]. The present study extended our previous work in the utilization of the novel series of RDIL tasks in efficacy evaluation with two significant findings: one is that chronic TLJN (0.9 and 1.8 g/kg, p.o.) treatment improved cognition on RDIL in Aβ_1–40_ manipulated rats; the other is that its therapeutic effects could attribute to the enhancement of memory-related molecules in the hippocampus.

Reward-guided instrumental conditioning is controlled by two memory systems: a goal-directed process and a stimulus–response habit mechanism that involves two forms of learning. The first consists of establishing incentive by introduction to the reward, whereas the second consists of making an association between the response and receiving the reward [32]. Moreover, according to the associative theory, instrumental learning is mediated by cues (stimulus) that predict the reward (outcome) and actions is learned (response) to gain access to the reward. The capacity for instrumental conditioning depends critically on the ability to encode a causal relationship among the three essential factors.

After all the rats received surgery, a goal-directed learning task and a habit learning task were successively conducted. Goal-directed actions are controlled by their consequences, habits are formed according to antecedent stimuli. Previous studies have shown that a higher rate of response schedule generates goal-directed actions while habitual, stimulus-driven action has been characterized by lower response rates [33,34]. Thus, a modified gradual higher rate of response schedule was firstly used to assess the cognitive flexibility. It is well known that animals can not only encode a causal relationship between an action and its consequence, but can also detect changes in the consequences of their actions. The results revealed that the Aβ_1–40_ injection prevented the renewal of A-O contingency when using the higher Fixed Ratio schedule but not the lower. Consistent with our previous findings, the Aβ_1–40_ group was insensitive to the contingency decline until the response ratio was raised to FR4 [6]. They were rendered unable to distinguish the consequences after a series of consecutive lever pressings and showed a profound and enduring loss of efficiency. Chronic administration of TLJN (0.9 and 1.8 g/kg, p.o.) increased the number of earned reward, the efficiency of operation and enhanced the perception of A-O association. Moreover, it made the rats maintain a persistent motivation which has been defined as the mapping between outcome and its value during goal-driven task [35,36]. However, the DNP (3 mg/kg, p.o.) group merely displayed a tendency to improve the cognitive flexibility when facing with the contingency degradation.

We further evaluated the stimulus-driven habit learning with a signal discrimination task. A deficiency in declarative memory is the main feature of the early stages of AD [37,38]. Declarative memories are reactivated by cues associated with the original acquisition of information [39]. Thereafter, they can provide detailed records of past experiences to guide goal-directed behavior [40]. The alternatively presented visual cue which implied a different task demand could be classified as contextual [41,42]. Accordingly, it could be applied in testing declarative memory as a form of external cue. Importantly, in present task, contextual discrimination was not aided by spatial cues but visual signal which based on pre-acquired habit memory [43]. Furthermore, during the decision-making process, actions are chosen by comparing their relative cached values, rather than their consequent outcomes. Thus, the reinforcement was no longer part of the S-R association, but simply maintained it [44]. It is evident that the hippocampus is crucial for tasks that involve forming causal relation among cues and consequences [45], whereas Aβ_1–40_ disrupts this function. Our findings demonstrated that chronic administration of TLJN (0.9 and 1.8 g/kg, p.o.) and DNP (3 mg/kg, p.o.) ameliorated the Aβ_1–40_-induced disability to encode a causal association between the visual cue and response. Thus, it was conceivable that the drugs treatment improved the ability to process contextual information which the rats utilized to adjust their responses to the proper cues reflecting the correct association with consequences.

In addition to these, the results obtained in extinction test further revealed a similar stimulus-driven memory process. Obviously, the test was carried out in the absence of the reinforcer to probe the nature of the memory. Several lines of evidence indicated that extinction involved previously learned associations without contamination by new learning rather than simply forgetting or erasure of original learning [35,46]. So far at the time, the rats had encoded the specific S-R association and were able to utilize it to guide behavior. Although all subjects were resistant to extinction during the test phase [47], the lever pressing and nose poke activities were gradually diminished. Our findings highlighted the role of failure in suppressing the non-rewarded response in the Aβ_1–40_-induced signal discrimination deficit. The TLJN (1.8 g/kg, p.o.) and DNP (3 mg/kg, p.o.) treated rats exhibited a better memory of the relationship between the visual signal and the specific response without the interference of the reward substances.

As we mentioned, Aβ plays a primary role in the pathogenesis of AD. Its accumulation is associated with hippocampal network dysfunction and results in cognitive deficits [48]. An in vivo hippocampal microinjection of Aβ in rodents induces neuronal dysfunction [49,50]. In accord with previous reports, we observed a disorder of cholinergic and glutamatergic neurotransmission, which are neurochemical characteristics of AD, in the hippocampus of Aβ_1–40_ model group [20,51]. It was accompanied with the inactivation of M1 receptors and over-expression of NR2B receptors. Cholinergic neurotransmission is essential for cue information processing and encoding [21,52]. And a decline in the Ach level was reported to interfere with the performance in lever press responses [53] and signal detection [54]. Ach signals by activating ligand-gated ion channels (nicotinic receptors) and metabotropic (muscarinic) G protein-coupled receptors [55]. Among the subtypes of cholinergic receptors, M1 receptors, predominant in forebrain and hippocampus, have a crucial role in regulating pathological process in AD [56-60]. Moreover, glutamate excitotoxicity is believed to be a mechanism contributing to progressive neuronal loss in AD. Aβ oligomers are reported to increase extracellular glutamate levels by interrupting glutamate reuptake and consequently inhibit NMDAR dependent hippocampal LTP principally as a result of over activation of NR2B-containing receptors [19,61]. Furthermore, alterations in membrane receptors activity can interfere with down-stream signaling pathway which is considered to be a crucial player in neuronal plasticity [62-64]. The ERK-1 and ERK-2 (ERK1/2) are the members of the mitogen activated protein kinase (MAPK) family and necessary for cell growth, differentiation, survival, molecular information processing and structural changes stabilizing in dendritic spines [56,65-68]. It is a highly conserved kinase cascade linking transmembrane receptors to downstream effector mechanisms [69]. CaMKII is the downstream effector of NMDA receptors and through which Aβdisrupt the dynamic balance in place between protein kinase and phosphatase presumed to be critical during neuronal plasticity [64,70]. Ultimately, neuronal development and plasticity are dependent on transcription of numerous genes. Multiple cell surface protein receptors triggered signal cascades converge on CREB leading to gene expression changes that are thought to be responsible for dendritic development and synapse formation [71-74]. Consistent with previous findings, our data clearly demonstrated that soluble Aβ_1–40_ disrupted the activation of ERK [75], CaMKII [70] and consequently inhibited CREB phosphorylation [76]. Liu et al. reported that TLJN could promote the degrading of Aβ and elimination of amyloid plaque in both the hippocampus and cortex via up-regulating insulin-degrading enzyme and neprilys in levels [24]. Correspondingly, our results evidently showed that TLJN (0.9 and 1.8 g/kg, p.o.) could not only restore the deficiency in cholinergic and glutamatergic neurotransmission, but also enhance the expression of downstream signal transduction molecules, such as ERK, CaMKII, CREB, in the hippocampus. Therefore, considering the functional role of these molecules in regulating learning and memory, the modulation of ERK/CaMKII/CREB signaling transduction could account for the therapeutic effect of TLJN.

In addition, DNP is one of the prescribed acetylcholinesteraseinhibitor drugs. It has been used as the first-line therapies to treat the dementia symptoms of AD patient via inhibition of acetylcholinesterase in the brain [77]. Intriguingly, DNP showed a limited extent in improving cognitive impairment in goal-directed behavior, but a significant ameliorating effect in habitual learning in present study. In consideration of the fact that multiple neural signaling pathways participate in regulating adaptive behavior [6], it could be inferred that cholinergic and glutaminergic system play a more crucial role in habitual learning than goal-directed behavior regulation. This inference might partially explain the distinguishing efficiency of DNP displayed in these two forms of associative learning. Nevertheless, more detailed differences between regulating goal-directed behavior and habitual learning still requires further research. In addition, the results also indicated that multiple targeting strategies should be taken into consideration when searching for new treatment for AD.

## Conclusions

Our data demonstrated that TLJN improved the Aβ_1–40_-induced cognitive deficits in adaptive behavior. Moreover, its ameliorating effects could attribute to the modulation of ERK/CaMKII/CREB signaling in the hippocampus. Inconsideration of its significant therapeutic potency, we conclude that TLJN has the potential, possibly as complementary and alternative therapy, to prevent and/or delay the deterioration of cognitive impairment in AD.

## Abbreviations

Ach: Acetylcholine
Aβ: β-Amyloid
AD: Alzheimer’s disease
CREB: cAMP response element-binding protein
CaMKII: Ca2+/calmodulin-dependent protein kinase II
DNP: Donepezil hydrochloride
ERK: Extracellular signal-regulated kinase
Glu: Glutamate
LTP: Long term potentiation
mAChR: Muscarinic acetylcholine receptors
NMDA: N-methyl-D-aspartic acid receptors
RDIL: Reward-directed instrumental learning
TLJN: Tong Luo Jiu Nao

## References

[1] Blennow K, de Leon MJ, Zetterberg H (2006). Alzheimer’s disease. Lancet.

[2] Cummings JL (2004). Alzheimer’s disease. N Engl J Med.

[3] Albert MS (2011). Changes in cognition. Neurobiol Aging.

[4] Droes RM, van der Roest HG, van Mierlo L, Meiland FJ (2011). Memory problems in dementia: adaptation and coping strategies and psychosocial treatments. Expert Rev Neurother.

[5] Wimo A, Jonsson L, Bond J, Prince M, Winblad B (2013). The worldwide economic impact of dementia 2010. Alzheimers Dement.

[6] Shi Z, Sun X, Liu X, Chen S, Chang Q, Chen L (2012). Evaluation of an Abeta (1–40)-induced cognitive deficit in rat using a reward-directed instrumental learning task. Behav Brain Res.

[7] Shi Z, Chen L, Li S, Chen S, Sun X, Sun L (2013). Chronic scopolamine-injection-induced cognitive deficit on reward-directed instrumental learning in rat is associated with CREB signaling activity in the cerebral cortex and dorsal hippocampus. Psychopharmacology (Berl).

[8] Brembs B, Lorenzetti FD, Reyes FD, Baxter DA, Byrne JH (2002). Operant reward learning in Aplysia: neuronal correlates and mechanisms. Science.

[9] D’Aquila PS (2010). Dopamine on D2-like receptors “reboosts” dopamine D1-like receptor-mediated behavioural activation in rats licking for sucrose. Neuropharmacology.

[10] Kandel ER (2001). The molecular biology of memory storage: a dialogue between genes and synapses. Science.

[11] West EA, Forcelli PA, Murnen A, Gale K, Malkova L (2011). A visual, position-independent instrumental reinforcer devaluation task for rats. J Neurosci Methods.

[12] Selkoe DJ (2002). Alzheimer’s disease is a synaptic failure. Science.

[13] Haass C, Selkoe DJ (2007). Soluble protein oligomers in neurodegeneration: lessons from the Alzheimer’s amyloid beta-peptide. Nat Rev Mol Cell Biol.

[14] Shankar GM, Bloodgood BL, Townsend M, Walsh DM, Selkoe DJ, Sabatini BL (2007). Natural oligomers of the Alzheimer amyloid-beta protein induce reversible synapse loss by modulating an NMDA-type glutamate receptor-dependent signaling pathway. J Neurosci.

[15] Miguel-Hidalgo JJ, Cacabelos R (1998). Beta-amyloid (1–40)-induced neurodegeneration in the rat hippocampal neurons of the CA1 subfield. Acta Neuropathol.

[16] Selkoe DJ (2001). Alzheimer’s disease: genes, proteins, and therapy. Physiol Rev.

[17] Lesne S, Koh MT, Kotilinek L, Kayed R, Glabe CG, Yang A (2006). A specific amyloid-beta protein assembly in the brain impairs memory. Nature.

[18] Walsh DM, Klyubin I, Fadeeva JV, Cullen WK, Anwyl R, Wolfe MS (2002). Naturally secreted oligomers of amyloid beta protein potently inhibit hippocampal long-term potentiation in vivo. Nature.

[19] Li S, Hong S, Shepardson NE, Walsh DM, Shankar GM, Selkoe D (2009). Soluble oligomers of amyloid Beta protein facilitate hippocampal long-term depression by disrupting neuronal glutamate uptake. Neuron.

[20] Langmead CJ, Watson J, Reavill C (2008). Muscarinic acetylcholine receptors as CNS drug targets. Pharmacol Ther.

[21] Hasselmo ME (2006). The role of acetylcholine in learning and memory. Curr Opin Neurobiol.

[22] Ma QL, Harris-White ME, Ubeda OJ, Simmons M, Beech W, Lim GP (2007). Evidence of Abeta- and transgene-dependent defects in ERK-CREB signaling in Alzheimer’s models. J Neurochem.

[23] Snyder EM, Nong Y, Almeida CG, Paul S, Moran T, Choi EY (2005). Regulation of NMDA receptor trafficking by amyloid-beta. Nat Neurosci.

[24] Liu Y, Hua Q, Lei H, Li P (2011). Effect of Tong Luo Jiu Nao on Abeta-degrading enzymes in AD rat brains. J Ethnopharmacol.

[25] Hua Q, Qing X, Li P, Li W, Hou J, Hu J (2010). Brain microvascular endothelial cells mediate neuroprotective effects on ischemia/reperfusion neurons. J Ethnopharmacol.

[26] Wang J, Li PT, Du H, Hou JC, Li WH, Pan YS (2012). Tong Luo Jiu Nao injection, a traditional Chinese medicinal preparation, inhibits MIP-1beta expression in brain microvascular endothelial cells injured by oxygen-glucose deprivation. J Ethnopharmacol.

[27] Paxinos GWC (1998). The rat brain in stereotaxic coordinates.

[28] Lansink CS, Goltstein PM, Lankelma JV, McNaughton BL, Pennartz CM (2009). Hippocampus leads ventral striatum in replay of place-reward information. PLoS Biol.

[29] Luu TT, Pirogovsky E, Gilbert PE (2008). Age-related changes in contextual associative learning. Neurobiol Learn Mem.

[30] Oler JA, Markus EJ (2000). Age-related deficits in the ability to encode contextual change: a place cell analysis. Hippocampus.

[31] Rand-Giovannetti E, Chua EF, Driscoll AE, Schacter DL, Albert MS, Sperling RA (2006). Hippocampal and neocortical activation during repetitive encoding in older persons. Neurobiol Aging.

[32] Balleine BW, Dickinson A (1998). Goal-directed instrumental action: contingency and incentive learning and their cortical substrates. Neuropharmacology.

[33] Dickinson A, Nicholas DJ, Adams CD (1983). The effect of the instrumental training contingency on susceptibility to reinforcer devaluation. J Exp Psychology.

[34] Dickinson A, Balleine B, Eilan N, McCarthy RA (1993). Actions and responses: The dual psychology of behaviour. Spatial representation: problems in philosophy and psychology.

[35] Niv Y, Joel D, Dayan P (2006). A normative perspective on motivation. Trends Cogn Sci.

[36] Balleine BW (2005). Neural bases of food-seeking: affect, arousal and reward in corticostriatolimbic circuits. Physiol Behav.

[37] Auld DS, Kornecook TJ, Bastianetto S, Quirion R (2002). Alzheimer’s disease and the basal forebrain cholinergic system: relations to beta-amyloid peptides, cognition, and treatment strategies. Prog Neurobiol.

[38] Sabe L, Jason L, Juejati M, Leiguarda R, Starkstein SE (1995). Dissociation between declarative and procedural learning in dementia and depression. J Clin Exp Neuropsychol.

[39] Sara SJ (2000). Retrieval and reconsolidation: toward a neurobiology of remembering. Learn Mem.

[40] Kennedy PJ, Shapiro ML (2009). Motivational states activate distinct hippocampal representations to guide goal-directed behaviors. Proc Natl Acad Sci U S A.

[41] Kennedy PJ, Shapiro ML (2004). Retrieving memories via internal context requires the hippocampus. J Neurosci.

[42] White NM, McDonald RJ (2002). Multiple parallel memory systems in the brain of the rat. Neurobiol Learn Mem.

[43] Broadbent NJ, Squire LR, Clark RE (2007). Rats depend on habit memory for discrimination learning and retention. Learn Mem.

[44] Yin HH, Knowlton BJ (2006). The role of the basal ganglia in habit formation. Nat Rev Neurosci.

[45] Lovinger DM (2010). Neurotransmitter roles in synaptic modulation, plasticity and learning in the dorsal striatum. Neuropharmacology.

[46] Nag S, Tang F, Yee BK (2001). Chronic intracerebroventricular exposure to beta-amyloid (1–40) impairs object recognition but does not affect spontaneous locomotor activity or sensorimotor gating in the rat. Exp Brain Res.

[47] Williams JH, Gray JA, Sinden J, Buckland C, Rawlins JN (1990). Effects of GABAergic drugs, fornicotomy, hippocampectomy and septal lesions on the extinction of a discrete-trial fixed ratio 5 lever-press response. Behav Brain Res.

[48] Villette V, Poindessous-Jazat F, Bellessort B, Roullot E, Peterschmitt Y, Epelbaum J (2012). A new neuronal target for beta-amyloid peptide in the rat hippocampus. Neurobiol Aging.

[49] Cantarella G, Uberti D, Carsana T, Lombardo G, Bernardini R, Memo M (2003). Neutralization of TRAIL death pathway protects human neuronal cell line from beta-amyloid toxicity. Cell Death Differ.

[50] Han M, Liu Y, Tan Q, Zhang B, Wang W, Liu J (2011). Therapeutic efficacy of stemazole in a beta-amyloid injection rat model of Alzheimer’s disease. Eur J Pharmacol.

[51] Levey AI (1996). Muscarinic acetylcholine receptor expression in memory circuits: implications for treatment of Alzheimer disease. Proc Natl Acad Sci U S A.

[52] Hasselmo ME, Stern CE (2006). Mechanisms underlying working memory for novel information. Trends Cogn Sci.

[53] Iso H, Ueki A, Shinjo H, Miwa C, Morita Y (1999). Reinforcement enhances hippocampal acetylcholine release in rats: an in vivo microdialysis study. Behav Brain Res.

[54] Sarter M, Hasselmo ME, Bruno JP, Givens B (2005). Unraveling the attentional functions of cortical cholinergic inputs: interactions between signal-driven and cognitive modulation of signal detection. Brain Res Rev.

[55] Ma L, Seager MA, Wittmann M, Jacobson M, Bickel D, Burno M (2009). Selective activation of the M1 muscarinic acetylcholine receptor achieved by allosteric potentiation. Proc Natl Acad Sci U S A.

[56] Caccamo A, Oddo S, Billings LM, Green KN, Martinez-Coria H, Fisher A (2006). M1 receptors play a central role in modulating AD-like pathology in transgenic mice. Neuron.

[57] Jones CK, Brady AE, Davis AA, Xiang Z, Bubser M, Tantawy MN (2008). Novel selective allosteric activator of the M1 muscarinic acetylcholine receptor regulates amyloid processing and produces antipsychotic-like activity in rats. J Neurosci.

[58] Conn PJ, Jones CK, Lindsley CW (2009). Subtype-selective allosteric modulators of muscarinic receptors for the treatment of CNS disorders. Trends Pharmacol Sci.

[59] Wess J, Eglen RM, Gautam D (2007). Muscarinic acetylcholine receptors: mutant mice provide new insights for drug development. Nat Rev Drug Discov.

[60] Davis AA, Fritz JJ, Wess J, Lah JJ, Levey AI (2010). Deletion of M1 muscarinic acetylcholine receptors increases amyloid pathology in vitro and in vivo. J Neurosci.

[61] Li S, Jin M, Koeglsperger T, Shepardson NE, Shankar GM, Selkoe DJ (2011). Soluble Abeta oligomers inhibit long-term potentiation through a mechanism involving excessive activation of extrasynaptic NR2B-containing NMDA receptors. J Neurosci.

[62] Adams JP, Sweatt JD (2002). Molecular psychology: roles for the ERK MAP kinase cascade in memory. Annu Rev Pharmacol Toxicol.

[63] Benito E, Barco A (2010). CREB’s control of intrinsic and synaptic plasticity: implications for CREB-dependent memory models. Trends Neurosci.

[64] Wayman GA, Lee YS, Tokumitsu H, Silva AJ, Soderling TR (2008). Calmodulin-kinases: modulators of neuronal development and plasticity. Neuron.

[65] Ivanov A, Pellegrino C, Rama S, Dumalska I, Salyha Y, Ben-Ari Y (2006). Opposing role of synaptic and extrasynaptic NMDA receptors in regulation of the extracellular signal-regulated kinases (ERK) activity in cultured rat hippocampal neurons. J Physiol.

[66] Berkeley JL, Gomeza J, Wess J, Hamilton SE, Nathanson NM, Levey AI (2001). M1 muscarinic acetylcholine receptors activate extracellular signal-regulated kinase in CA1 pyramidal neurons in mouse hippocampal slices. Mol Cell Neurosci.

[67] Lee YS, Silva AJ (2009). The molecular and cellular biology of enhanced cognition. Nat Rev Neurosci.

[68] Sindreu CB, Scheiner ZS, Storm DR (2007). Ca2+ − stimulated adenylyl cyclases regulate ERK-dependent activation of MSK1 during fear conditioning. Neuron.

[69] Kelleher RJ, Govindarajan A, Jung HY, Kang H, Tonegawa S (2004). Translational control by MAPK signaling in long-term synaptic plasticity and memory. Cell.

[70] Zhao D, Watson JB, Xie CW (2004). Amyloid beta prevents activation of calcium/calmodulin-dependent protein kinase II and AMPA receptor phosphorylation during hippocampal long-term potentiation. J Neurophysiol.

[71] Athos J, Impey S, Pineda VV, Chen X, Storm DR (2002). Hippocampal CRE-mediated gene expression is required for contextual memory formation. Nat Neurosci.

[72] Impey S, Obrietan K, Wong ST, Poser S, Yano S, Wayman G (1998). Cross talk between ERK and PKA is required for Ca2+ stimulation of CREB-dependent transcription and ERK nuclear translocation. Neuron.

[73] Tully T, Bourtchouladze R, Scott R, Tallman J (2003). Targeting the CREB pathway for memory enhancers. Nat Rev Drug Discov.

[74] Hardingham GE, Fukunaga Y, Bading H (2002). Extrasynaptic NMDARs oppose synaptic NMDARs by triggering CREB shut-off and cell death pathways. Nat Neurosci.

[75] Townsend M, Mehta T, Selkoe DJ (2007). Soluble Abeta inhibits specific signal transduction cascades common to the insulin receptor pathway. J Biol Chem.

[76] Scott Bitner R (2012). Cyclic AMP response element-binding protein (CREB) phosphorylation: a mechanistic marker in the development of memory enhancing Alzheimer’s disease therapeutics. Biochem Pharmacol.

[77] Relkin NR (2007). Beyond symptomatic therapy: a re-examination of acetylcholinesterase inhibitors in Alzheimer’s disease. Expert Rev Neurother.

